# Use of Baricitinib in a patient with treatment-resistant pyoderma gangrenosum

**DOI:** 10.1177/2050313X241235444

**Published:** 2024-03-23

**Authors:** Kaylin Bechard, Robert Gniadecki

**Affiliations:** Department of Dermatology, University of Alberta Medicine, AB, Canada

**Keywords:** Immunology, inflammatory dermatoses, wound

## Abstract

Pyoderma gangrenosum is a rare inflammatory neutrophilic disorder with no uniformly effective therapy and limited high-level evidence. Common therapies include immunosuppressive and immunomodulating agents. There exist several case series using small molecules as treatment modalities. Here, we report a case of a 78-year-old female with a diagnosis of pyoderma gangrenosum and metastatic high-grade serous carcinoma of the ovary who was treated with Baricitinib 4 mg daily in combination with a tapering course of prednisone after failing other conventional therapies including systemic corticosteroids, colchicine, and intravenous immunoglobulin.

## Introduction

Pyoderma gangrenosum (PG) is a rare inflammatory neutrophilic disorder that classically presents as a painful, rapidly evolving cutaneous ulcers with undermined, irregular, erythematous-violaceous edges.^1–4^ Management is challenging as treatment requires reducing the inflammatory process of the wound to promote healing.^1,2^ There is no uniformly effective therapy and high-level evidence is limited.^2,3^ It is most often treated with immunosuppressive and immunomodulating agents such as corticosteroids, cyclosporine, mycophenolate mofetil, dapsone, intravenous immunoglobulin (IVIg), and biologic therapy,^5^ namely tumor necrosis factor (TNF)-α inhibitors and interleukin (IL)-1β antagonists.^3,4^ Other treatment options include the use of small molecules, for which there exist case several case series.^4^ Baricitinib, a Janus kinase (JAK)-1 and JAK-2 inhibitor, has led to complete ulcer healing in isolated reports.^6,7^ A phase II clinical trial (NCT04901325) using Baricitinib in combination with prednisone is currently on hold.^8^ Here, we report a case of PG treated with Baricitinib 4 mg daily in combination with a tapering course of prednisone.

## Case report

A 78-year-old female presented to dermatology after developing a painful ulcerative lesions in the right axilla, right elbow, and left forearm that continued to increase in size. She carried a medical history of metastatic high-grade serous carcinoma of the ovary, for which she had completed palliative-intent chemotherapy and was on weekly paclitaxel. Her remaining medical history included previous pulmonary embolus, dyslipidemia, and gastroesophageal reflux disease.

A biopsy of the left forearm showed a central erosion with heavy surface acute inflammatory exudate, subepithelial perivascular and interstitial lymphocytic infiltration with occasional admixed neutrophils. Eosinophils were not a significant feature. A clinical diagnosis of PG was suspected and she was treated empirically over a 6 month period with oral and intralesional corticosteroids, minocycline, and wound care. There was minimal to no improvement.

Due to her refractory ulcerations and suspected PG, she was referred to a tertiary care outpatient clinic for ongoing management. Two additional biopsies were taken, each of the right axilla. The first demonstrated the edge of an ulceration with dermal fibrosis, mixed inflammatory infiltrate in the deep reticular dermis and subcutis, and eosinophilic fragmented elastic fibers. The second demonstrated ulceration with neutrophilic infiltrate with fibrin, fibrosis, and chronic inflammatory changes in the dermis, with and fat necrosis in the subcutis. During this 5-month period, she was treated with systemic corticosteroids, colchicine, and ongoing wound care. This resulted in minimal improvement with her wounds remaining unchanged.

**Figure A1.**
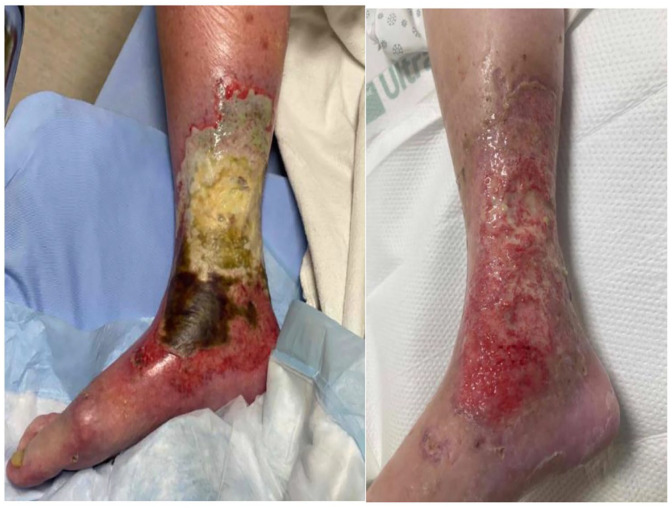
Right lower extremity wound. Left: Prior to treatment with Baricitinib. Right: Completion of 17 days of Baricitinib.

She then developed an additional ulcer on the right lower extremity following removal of an electrocardiogram lead electrode. This ulcer was painful and continued to increase in size (Figure A1). Moreover, the ulcers on her right axilla (Figure A6) and right elbow (Figure A4) had also increased in size over this time, and demonstrated perilesional erythema. Her paclitaxel was held. She subsequently received two cycles of IVIg to treat her refractory PG.

Due to the evolving nature of the right lower extremity ulcer and secondary infection, she was admitted to hospital for ongoing management. Physical examination showed an unstageable ulcer covered by necrotic tissue involving the medial aspect of the right lower leg, ankle, and foot (Figure A1). She was treated with antimicrobial therapy for her secondary polymicrobial infection and underwent surgical debridement as an inpatient. She had no evidence of myelodysplasia, hematologic malignancy, or inflammatory bowel disease. Any additional therapeutic options were limited due to her known metastatic high-grade serous carcinoma of the ovary. Furthermore, treatment of her malignancy was held due to her worsening PG. Due to these factors and failed treatments, the patient was started on Baricitinib 4 mg daily in combination with a tapering course of prednisone.

**Figure A2.**
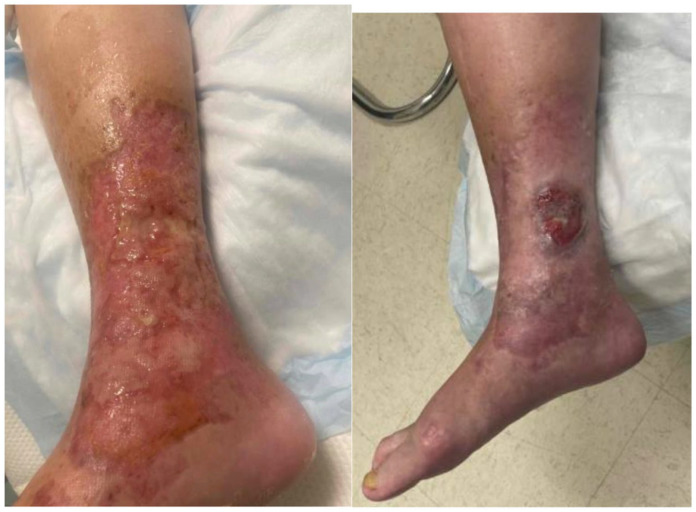
Right lower extremity wound. Left: Right: Completion of 5 weeks of Baricitinib. Right: 4 weeks after discontinuing Baricitinib (12 weeks completed).

A total of 12 weeks of therapy with Baricitinib was completed. During this time, the right lower extremity ulcer decreased in size (Figures A1 and A2). The ulcers on the right axilla (Figure A6) and right elbow (Figure A4) resolved. Given this, her systemic corticosteroid therapy was tapered.

**Figure A3.**
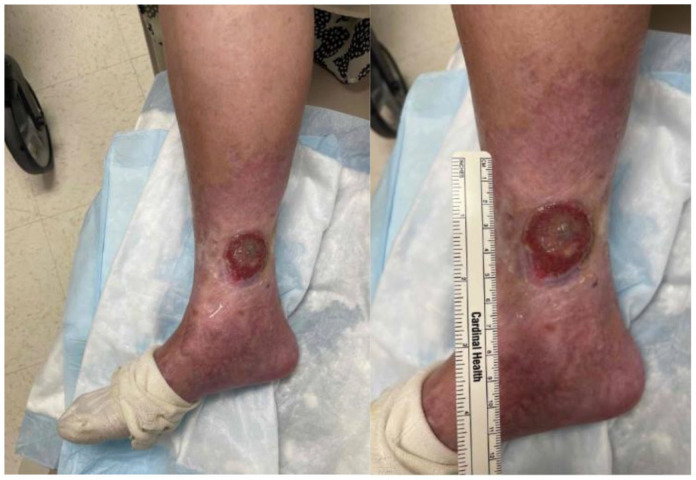
Right lower extremity wound. Left, Right: 13 weeks after discontinuing Baricitinib.

**Figure A4.**
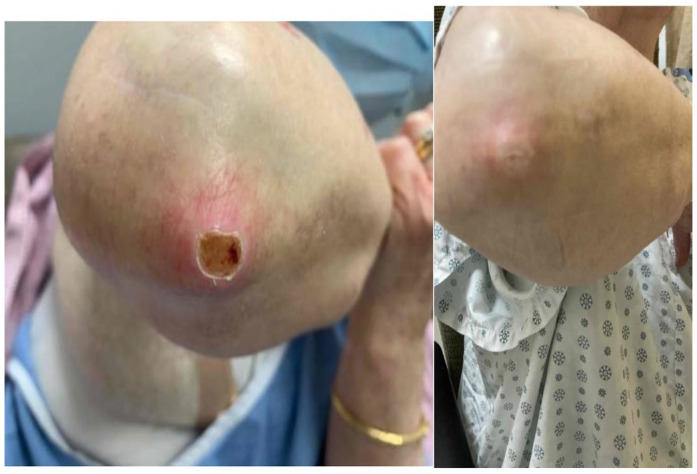
Right elbow. Left: Prior to treatment with Baricitinib. Right: Completion of 3 weeks of Baricitinib.

**Figure A5.**
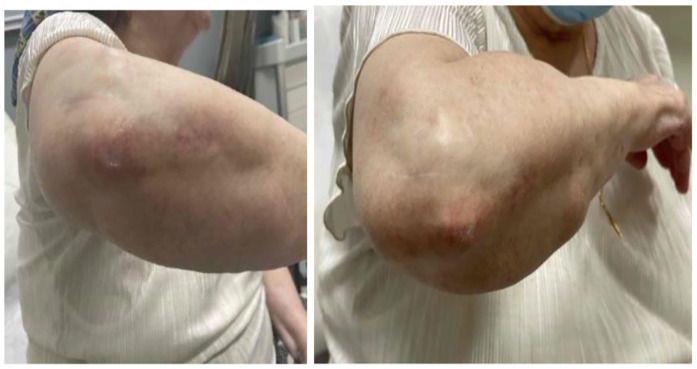
Right elbow. Left: 4 weeks after discontinuing Baricitinib (12 weeks completed). Right: 13 weeks after discontinuing Baricitinib.

**Figure A6.**
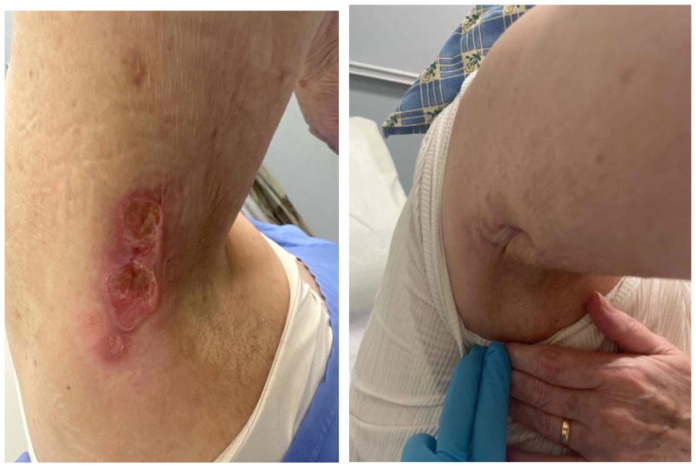
Right axilla. Left: Prior to treatment with Baricitinib. Right: 4 weeks after discontinuing Baricitinib (12 weeks completed).

**Figure A7.**
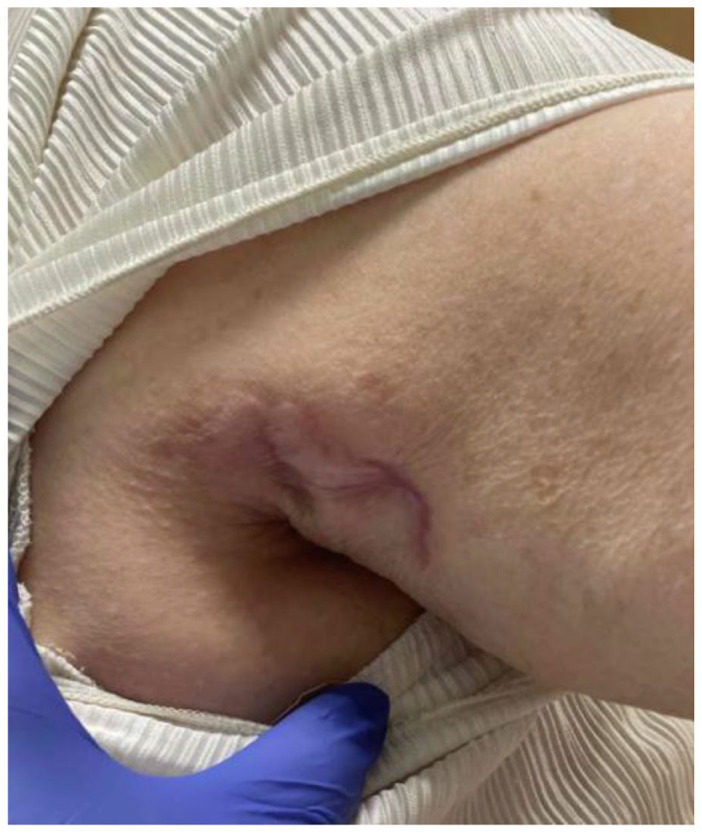
Right axilla. Right: 13 weeks after discontinuing Baricitinib.

Following this, the Baricitinib was discontinued at the discretion of the patient and their oncology team when weekly paclitaxel was resumed for ongoing palliative treatment of her high-grade serous carcinoma of the ovary. Shortly thereafter, she was admitted to hospital after developing methicillin-sensitive *Staphylococcus aureus* right knee native joint septic arthritis without bacteremia, managed with treated with orthoscopic washout and antimicrobial therapy. After her recent admission, 4 weeks had elapsed since she had discontinued the Baricitinib. The ulcer on the right lower extremity had recurred (Figures A2 and A3). The ulcers on the her right axilla and right elbow did not recur (Figures A5 and A7). At her last outpatient visit, 13 weeks after discontinuing the Baricitinib, the right axilla and right elbow ulcers did not recur. The right lower extremity ulcer further progressed (Figure A3).

## Discussion

The pathophysiology of PG remains incompletely understood, it has a multifactorial pathogenesis, including dysregulation of both innate and adaptive immunity, abnormal chemotaxis, neutrophil migration, phagocytosis, bactericidal ability, and abnormal neutrophil trafficking.^7,8^ Elevated levels of TNF-α, IL-1β, IL-1α, IL-8, IL-12, IL-15, IL-17, IL-23, and IL-36 have been identified in a self-maintaining, autoinflammatory milieu.^2,4^

The JAK/signal transducer and activator of transcription (JAK/STAT) signaling pathway is regarded as one of the central communication nodes in the cell function, regulating multiple cellular mechanisms associated with disease development in inflammatory disease.^9^ Targeting therapies suppress JAK function cause immunosuppression and decrease the abnormally elevated serum proinflammatory cytokines mediated by the JAK/STAT signaling pathway.^9^ Cytokines upstream of JAK/STAT signaling include ILs 1–31, TNF-α, and colony-stimulating factors, among others.^4^ JAK activation results in phosphorylation of specific STAT proteins.^4^ When activated, STAT proteins translocate to the nucleus and stimulate or inhibit the transcription of genes involved in hematopoiesis and immune function.^4^

The JAK/STAT pathway has been shown to be dysregulated in PG lesions with JAK-1, JAK-2, and JAK-3 and STAT1 through six being overexpressed in the skin of patients with PG compared with healthy controls.^4^ Mutations in the JAK/STAT pathway are also implicated in PG progression.^4^ JAK inhibitors that have been used for the treatment of PG include tofacitinib (JAK-1, JAK-2), ruxolitinib (JAK-1, JAK-2), and baricitinib (JAK-1, JAK-2).^4^ Our case, along with other isolated reports,^6,7^ shows that Baricitinib may be a promising therapy in refractory PG where other immunosuppressive and immunomodulating agents fail or are contraindicated.
